# Proteomic analysis of canine oral tumor tissues using MALDI-TOF mass spectrometry and in-gel digestion coupled with mass spectrometry (GeLC MS/MS) approaches

**DOI:** 10.1371/journal.pone.0200619

**Published:** 2018-07-12

**Authors:** Sirinun Pisamai, Sittiruk Roytrakul, Narumon Phaonakrop, Janthima Jaresitthikunchai, Gunnaporn Suriyaphol

**Affiliations:** 1 Biochemistry Unit, Department of Physiology, Faculty of Veterinary Science, Chulalongkorn University, Bangkok, Thailand; 2 Companion Animal Cancer Research Unit, Faculty of Veterinary Science, Chulalongkorn University, Bangkok, Thailand; 3 Proteomics Research Laboratory, Genome Institute, National Center for Genetic Engineering and Biotechnology, National Science and Technology Development Agency, Pathum Thani, Thailand; University of Maryland School of Medicine, UNITED STATES

## Abstract

Oral tumors, including highly invasive and metastatic oral melanoma (OM), non-tonsillar oral squamous cell carcinoma (OSCC) and benign tumors (BN), are common neoplasms in dogs. Although these tumors behave differently, limited data of their protein expression profiles have been exhibited, particularly at the proteome level. The present study aimed to i.) characterize peptide-mass fingerprints (PMFs) and identify potential protein candidates of OM, OSCC, BN and normal control subjects, using matrix-assisted laser desorption/ionization time-of-flight mass spectrometry (MALDI-TOF MS) and liquid chromatography tandem mass spectrometry (LC-MS/MS), ii.) identify potential protein candidates associated with the diseases, using in-gel digestion coupled with mass spectrometric analysis (GeLC-MS/MS) and iii.) search for relationships between chemotherapy drugs and disease-perturbed proteins. A distinct cluster of each sample group and unique PMFs with identified protein candidates were revealed. The unique peptide fragment at 2,274 Da of sacsin molecular chaperone (SACS) was observed in early-stage OM whereas the fragment at 1,958 Da of sodium voltage-gated channel alpha subunit 10 (SCN10A) was presented in early- and late-stage OM. The peptide mass at 2,316 Da of Notch1 appeared in early-stage OM and benign oral tumors while the peptide mass at 2,505 Da of glutamate ionotropic receptor N-methyl-D-aspartate type subunit 3A (GRIN3A) was identified in all groups. Markedly expressed proteins from GeLC-MS/MS included Jumonji domain containing 1C (JMJD1C) in benign tumors, inversin (INVS) and rho guanine nucleotide exchange factor 28 (ARHGEF28) in OM, BTB domain-containing 16 (BTBD16) in OSCC, and protein tyrosine phosphatase non-receptor type 1 (PTPN1), BRCA2, DNA repair associated (BRCA2), WW domain binding protein 2 (WBP2), purinergic receptor P2Y1 and proteasome activator subunit 4 (PSME4) in all cancerous groups. The network connections between these proteins and chemotherapy drugs, cisplatin and doxorubicin, were also demonstrated. In conclusion, this study unveiled the unique PMFs and novel candidate protein markers of canine oral tumors.

## Introduction

Oral neoplasms represent approximately 7% of all types of tumors in dogs [1]. Among these, oral melanoma (OM) is the most aggressive, with high prevalence, accounting for 30–40% of all oral cancers [2, 3] or 15–45% of all oral tumors [4]. According to the World Health Organization (WHO) clinical staging scheme of OM, the prognosis is based on tumor size, lymph node involvement and distant metastasis or TNM system. Stage I is a tumor <2 cm in diameter; stage II is a 2 to <4 cm diameter tumor; stage III is a tumor ≥4 cm in diameter with or without lymph node metastasis, and stage IV is a tumor with distant metastasis [2]. Several cases of OM were detected at the late stages (stages III and IV) with poor prognosis [5, 6]. OM cases generally recurred and/or metastasized rapidly after surgical resection [7]. Oral squamous cell carcinoma (OSCC) is another common oral neoplasm in dogs, comprising 20–30% of all oral tumors [8]. The frequently found non-tonsillar type is less aggressive than the tonsillar one [7]. The histological grading of OSCC is defined as well, moderately and poorly differentiated [9]. Well-differentiated SCC is similar to normal squamous epithelium, with compact laminated keratin or keratin pearls. For moderately and poorly differentiated SCC, greater degrees of mitotic activity and nuclear pleomorphism with less keratinization are present [9]. For the benign tumors (BN), the non-malignant lesions are classified according to the origin of the cells, including peripheral odontogenic fibroma (fibromatous epulis of periodontal ligament origin), acanthomatous ameloblastoma (epithelial neoplasia of the enamel organ), odontoma (odontogenic neoplasm of mixed origin) and other odontogenic tumors [8]. Several proteins have been reported to be potential biomarkers or therapeutic targets in canine oral tumors, such as melanoma cell adhesion molecule, cluster of differentiation 146 (CD146), Ras GTPase-activating-like protein IQGAP1, programmed death ligand-1, leptin, fascin-1, chondroitin sulfate proteoglycan-4 in OM, proliferating cell nuclear antigen, p63 and E-cadherin, high mobility group A2 in OSCC, and CD46 in ameloblastoma [10–19]. However, our knowledge of protein expression involved in the development and progression of canine oral tumors is still limited, particularly in a large-scale analysis. Proteomics is the study of expressed proteins under a specific condition in large scale [20]. Mass spectrometry (MS) is the high-throughput technology for protein profiling. A matrix-assisted laser desorption/ionization time-of-flight (MALDI-TOF), composed of a MALDI source and a TOF mass analyzer, is used for searching peptide mass fingerprints (PMFs). MS spectra are obtained and compared to get fingerprints of ions that are characteristic of the cell/tissue/organism. In addition, three-dimensional principal component analysis (3D PCA) scatterplot has been used to reveal the uniformity and homogeneity of the sample group [21]. MALDI-TOF MS was demonstrated as a rapid screening method to differentiate oral cancer, oral lichen planus, and chronic periodontitis in human saliva [22]. In dog, MALDI-TOF was used to study different protein expression in tears from dogs with cancers (transmissible venereal tumor, mammary gland adenocarcinoma, squamous cells carcinoma, fibrosarcoma, etc.) and normal dogs in order to develop tear film analysis for cancer diagnosis and management in dogs [23]. Specific mass spectra peaks on the PMF map can be further analyzed using MALDI-TOF/TOF MS, which was used to identify protein biomarkers in canine lymphoma, mammary tumor, prostate tumor and mast cell tumor [24–31]. Another tandem MS, liquid chromatography-tandem mass spectrometry (LC-MS/MS), is used for routine identification of proteins. LC-MS/MS use electrospray ionization (ESI) whereas MALDI-TOF uses MALDI as an ionization source with different operation and performance characteristics [32]. In-gel digestion coupled with mass spectrometric analysis (GeLC-MS/MS) is a one-dimensional sodium dodecyl sulfate-polyacrylamide gel electrophoresis (SDS-PAGE) followed by in-gel digestion and LC-MS/MS. GeLC-MS/MS is suitable for qualitative and quantitative complex protein identification [33]. In dogs, this method was used to compare protein expression of formalin-fixed paraffin embedded and fresh-frozen sets of the same tissues [34]. In addition, LC-MS/MS was used to measure plasma free metanephrine and free normetanephrine in dogs with pheochromocytoma for disease diagnosis and in lymphoma [30, 35]. There remain gaps in our knowledge of protein expression profiles of canine oral tumors, particularly at the proteome level.

Since OM has high potential to metastasize, a combination of treatments is usually required for the late-stage OM, clinical stages III and IV. A number of chemotherapy drugs have been used to cure canine oral cancers after surgery. At the animal teaching hospital, Faculty of Veterinary Science, Chulalongkorn University, dogs with the late-stage OM undergo carboplatin chemotherapy with the median dosage 300 mg/m^2^ for 6 times or else the metronomic therapy, continuous administration of low doses cyclophosphamide (10 mg/m^2^) and standard dose piroxicam (0.3 mg/kg) as previously reported [36, 37]. Carboplatin, a derivative of the anti-cancer drug cisplatin, and doxorubicin (also called adriamycin) are common chemotherapy drugs used in canine oral cancer treatment, whereas cyclophosphamide and piroxicam have also been widely used in metronomic chemotherapy [36, 38, 39]. In the present study, a fast and inexpensive bioinformatic tool was used to uncover functional relationships between drugs and disease proteins and fulfill experimental data. The proposed network maps demonstrated the molecular basis of disease, which could probably help select potential targets for early diagnosis, prognosis or effective treatment. The present study aimed to characterize PMFs of OM, OSCC, BN and normal control subjects, using MALDI-TOF and liquid chromatography tandem mass spectrometry (LC-MS/MS), to identify potential protein candidates associated with the diseases, using GeLC-MS/MS and to search for relationships between chemotherapy drugs and disease-perturbed proteins. Herein, we found a distinct cluster and a unique PMF in each canine oral tumor group. In addition, unique peptide fragment at 2,274 Da of sacsin molecular chaperone (SACS) was observed in early-stage OM, and at 1,958 Da of sodium voltage-gated channel alpha subunit 10 (SCN10A) in early- and late-stage OM. The peptide mass at 2,316 Da of Notch1 appeared in early-stage OM and benign oral tumors while the peptide mass at 2,505 Da of glutamate ionotropic receptor N-methyl-D-aspartate type subunit 3A (GRIN3A) was identified in all sample groups. We also found a peptide fragment at 3,039 Da of leucine-tRNA synthetase (LARS) in early-stage OM, OSCC, and benign oral tumors. Using GeLC-MS/MS, we discovered potentially novel candidate markers of canine oral tumors such as Jumonji domain containing 1C (JMJD1C or TRIP8) in benign tumors, inversin (INVS) and rho guanine nucleotide exchange factor 28 (ARHGEF28) in OM, BTB domain-containing 16 (BTBD16) in OSCC, and protein tyrosine phosphatase non-receptor type 1 (PTPN1), BRCA2, WW domain binding protein 2 (WBP2), purinergic receptor P2Y1 variant 2 (P2Y1) and proteasome activator subunit 4 (PSME4) in all cancerous groups. We also demonstrated relationships between cisplatin and doxorubicin and disease-perturbed proteins, whereas cyclophosphamide/piroxicam showed no or very faint relationships with most proteins. These data might help veterinarians choose drugs of choice and treatment plan.

## Materials and methods

### Sample collection

Tumor tissue samples were collected from patients undergoing surgery at the Small Animal Teaching Hospital, Faculty of Veterinary Science, Chulalongkorn University. They were comprised of 15 OM (7 early- and 8 late-stage OM), 7 OSCC and 8 benign oral tumors (age range 1–16 years). Eight normal gingiva tissue samples were collected from fresh dog carcasses with no history or clinical signs of oral cavity or cancerous problems (age range 8–9 years). OM was classified according to the TNM staging system of the WHO [2]. Stages I and II were defined as an early-stage OM, whereas stages III and IV were determined as the late-stage OM [40]. The sample collection protocol was approved by the Chulalongkorn University Animal Care and Use Committee (CU-ACUC) and samples were obtained with the consents of owners. Samples were bisected: one half was fixed in 10% neutral buffered formalin for histopathological diagnosis; and the other half was stored in RNAlater (Thermo Fisher Scientific, Waltham, MA) at –20°C for proteomic analysis, for which approximately 100 mg of tissues were pulverized in liquid nitrogen and incubated in 0.5% SDS for 1 h at room temperature, followed by centrifuging at 12,000 rpm for 15 min. Supernatants were kept at –20 °C until further analysis.

### Histopathology and immunohistochemistry

Formalin-fixed, paraffin-embedded (FFPE) sections of 4-μm thickness were stained with hematoxylin and eosin (H&E) for routine histopathological diagnosis. To confirm amelanotic melanoma, tissues were deparaffinized with xylene and rehydrated through a series of graded concentrations of ethanol in water. The sections were antigen retrieved in 0.01 M sodium citrate, pH 6.0, in a microwave oven (800 W) for 10 min. Endogenous peroxidase activity was quenched by incubating the slides in H_2_O_2_ 3% in methanol at room temperature for 10–20 min. Non-specific immunoglobulin binding was blocked with 1–3% (w/v) bovine serum albumin (Merck, Rockland, MA) at 37°C for 20 min. Sections were incubated with 1:50 mouse monoclonal against human Melan-A antibody (Dako, Glostrup, Denmark M7196), at 4 °C for 16 h. A polymer-based non-avidin–biotin system, the EnVision detection system system (Dako), was used for detection of the reaction, and labeling was visualized with a 3,3′-diaminobenzidine tetrahydrochloride (DAB) substrate kit (Dako). The sections were counterstained with Mayer’s hematoxylin. Canine melanotic melanoma was used as a positive control. The positive areas were seen in cytoplasmic areas.

### Analysis of peptide patterns by MALDI-TOF MS

Total protein concentrations were determined by Lowry’s assay at 690 nm, using bovine serum albumin as a standard [41]. Protein samples in each group were pooled, dried and redissolved in 100% acetonitrile (ACN) containing 5% trifluoroacetic acid (TFA). The samples of 1 μg/μL were mixed with MALDI matrix solution [10 mg/mL α-cyano-4-hydroxycinnamic acid (CHCA) in 100% ACN containing 5% TFA] at the ratio of 1:1, spotted as 20 individual replicates on to a MTP 384 ground steel target plate (Bruker Daltonics, Billerica, MA) and air dried. Mass spectra were acquired on the Ultraflex III TOF/TOF (Bruker Daltonics) in a linear positive mode over a mass range 1,000–20,000 Da. The standard peptide mixtures of ProteoMass Peptide & Protein MALDI-MS Calibration Kit (Sigma Aldrich, St. Louis, MO) were used for the external calibration, including human angiotensin II (m/z 1,046), P14R (m/z 1,533), human adrenocorticotropic hormone fragment 18–39 (m/z 2465), bovine insulin oxidized B chain (m/z 3,465), bovine insulin (m/z 5,731), and cytochrome c (m/z 12,362). Each spectrum was obtained from 500 laser shots, with a 50 Hz laser. Fingerprint spectra, pseudo-gel view and 3D PCA scatterplot were analyzed by ClinProTools version 3.0 and flexAnalysis version 3.3 software (Bruker Daltonics), respectively [22, 42]. To analyze candidate mass spectra between 1,000 and 20,000 Da, three statistical algorithms incorporated in the ClinProTools software version 3.0, including Quick Classifier (QC)/ Different Average, Supervised Neural Network (SNN) and the Genetic Algorithm (GA), were utilized. The recognition capability and cross validation values >90% were used to reveal the reliability of the candidate peak selection [43]. And in order to analyse intensity values, three statistical tests (Anderson-Darling (AD), t-test/ANOVA (TTA), and Wilcoxon/Kruskal-Wallis (W/KW), incorporated into ClinProTools software version 3.0, were used. Results with p<0.05 were considered significant. To analyze specific peptide sequences, peptide samples were purified using C18 ZipTip (Merck Millipore, Darmstadt, Germany) and eluted with 2% series of ACN. After that samples were analyzed by LC-MS/MS using an Ultimate 3000 LC System (Thermo Scientific Dionex, Waltham, MA, US) on a nanocolumn PepSwift monolithic column 100 mm i.d.650 mm, at a flow rate of 300 nL/min. The nanoLC system was connected to an electrospray interface with ESI-Ion Trap MS (Bruker Daltonics). The LC-MS raw data were converted into an mz XML file by CompassXport software (Bruker Daltonics). All data were obtained for quantification based on MS signal intensities of individual analysis using DeCyder MS differential analysis software (DeCyder MS, GE Healthcare, Amersham, UK). All MS/MS data from DeCyder MS software were submitted to a database search against the NCBI *Canis lupus familiaris* database using MASCOT software version 2.2 (Matrix Science, London, UK).

### Protein identification by GeLC-MS/MS

For the protein identification by GeLC-MS/MS, 50 μg of protein pools were fractionated on 12% SDS-PAGE (Atto, Tokyo, Japan). After Coomassie Brilliant Blue R-250 (CBB R-250) protein staining and destaining with 16.5% ethanol in 5% acetic acid, the gel was scanned using a GS-710 scanner (Bio-Rad, Benicia, CA) and stored in 0.1% acetic acid until in-gel tryptic digestion, where protein bands were divided into 25 segments per lane according to size and chopped into 1 mm^3^ pieces. Gel plugs were dehydrated using 100% ACN for 5 min and dried for 15 min at room temperature. Disulfide bonds were reduced by 10 mM dithiothreitol (DTT) in 10 mM ammonium bicarbonate for 1 h at room temperature and alkylated in 100 mM iodoacetamide (IAA) in 10 mM ammonium bicarbonate for 1 h at room temperature in the dark. The gel pieces were dehydrated twice in 100% ACN for 5 min and trypsin-digested overnight at 37 °C. The sequencing grade modified trypsin (Promega, Madison, WI) was used. The tryptic digestion was performed in 50mM NH_4_HCO_3_ (pH 7.8) as recommended by the manufacturer. The tryptic peptides were extracted from the gels using 50% ACN in 0.1% formic acid (FA). Finally, peptide mixtures were dried and kept at –80 °C until LC-MS/MS analysis.

Prior to sample injection into LC-MS/MS, dried extracted peptides were dissolved in 0.1% FA in LC/MS-grade water and centrifuged at 12,000 × *g* for 10 min. The peptide solutions were analyzed by an Ultimate 3000 LC System (Thermo Scientific Dionex, Sunnyvale, CA). PepSwift monolithic nanocolumn 100 mm i.d.650 mm at a flow rate of 300 nL/min, using a multi-step gradient of a 10–90% linear concentration of 80% ACN in 0.1% FA within 20 min. The nanoLC system was connected with an electrospray interface with ESI-Ion Trap MS (Bruker Daltonics). The LC-MS raw data were converted into an mz XML file by CompassXport software (Bruker Daltonics). All data were obtained for quantification based on MS signal intensities of individual analysis using DeCyder MS differential analysis software (DeCyder MS, GE Healthcare, Amersham, UK). ANOVA statistical analysis, incorporated into the DeCyder MS, was used to identify significantly varying peptides among different sample groups.

All MS/MS data from DeCyder MS software were submitted to a database search against the NCBI *Canis lupus familiaris* database using MASCOT software version 2.2 (Matrix Science, London, UK). Proteins were classified according to their molecular function, biological process and cellular component using PANTHER classification system version 8.1 [44]. Protein list comparison among different sample groups was displayed using jvenn diagram [45]. Proteins that were individually expressed in each group were chosen as candidate proteins. Then, the list of candidate proteins was analyzed for their relationship with carcinogenesis and chemotherapy drugs by the online-based software Stitch version 5.0 [46]. Hierarchical clustering heat map was performed using Multiexperiment Viewer (MeV) program, version 4.8, with the Pearson correlation distance metric [47]. The statistical significance level was set at p<0.05.

## Results

The histopathological classifications of the OM and samples are shown in Table 1 and S1 Fig. For the OSCC, 2 and 5 samples were diagnosed as poorly differentiated and well differentiated, respectively. The benign oral tumors comprised 4 acanthomatous ameloblastomas and 4 peripheral odontogenic fibromas.

**Table 1:** TNM and histopathological classifications of dogs with oral malignant melanoma.

Histological type	TNM Stages I–II*	TNM Stages III–IV*	Total
**Epithelioid, melanotic**	0	5	5/15 (%)
**Spindle, melanotic**	4	0	4/15 (%)
**Epithelioid, amelanotic**	2	3	5/15 (%)
**Spindle, amelanotic**	1	0	1/15 (%)
**Total**	7/15 (46.67%)	8/15 (53.33%)	15 (100%)

*TNM stage I is <2 cm diameter tumor, stage II is 2 cm to <4 cm diameter tumor, stage III is ≥ 4 cm tumor and/or lymph node metastasis and stage IV is tumor with distant metastasis.

**Fig 1:**
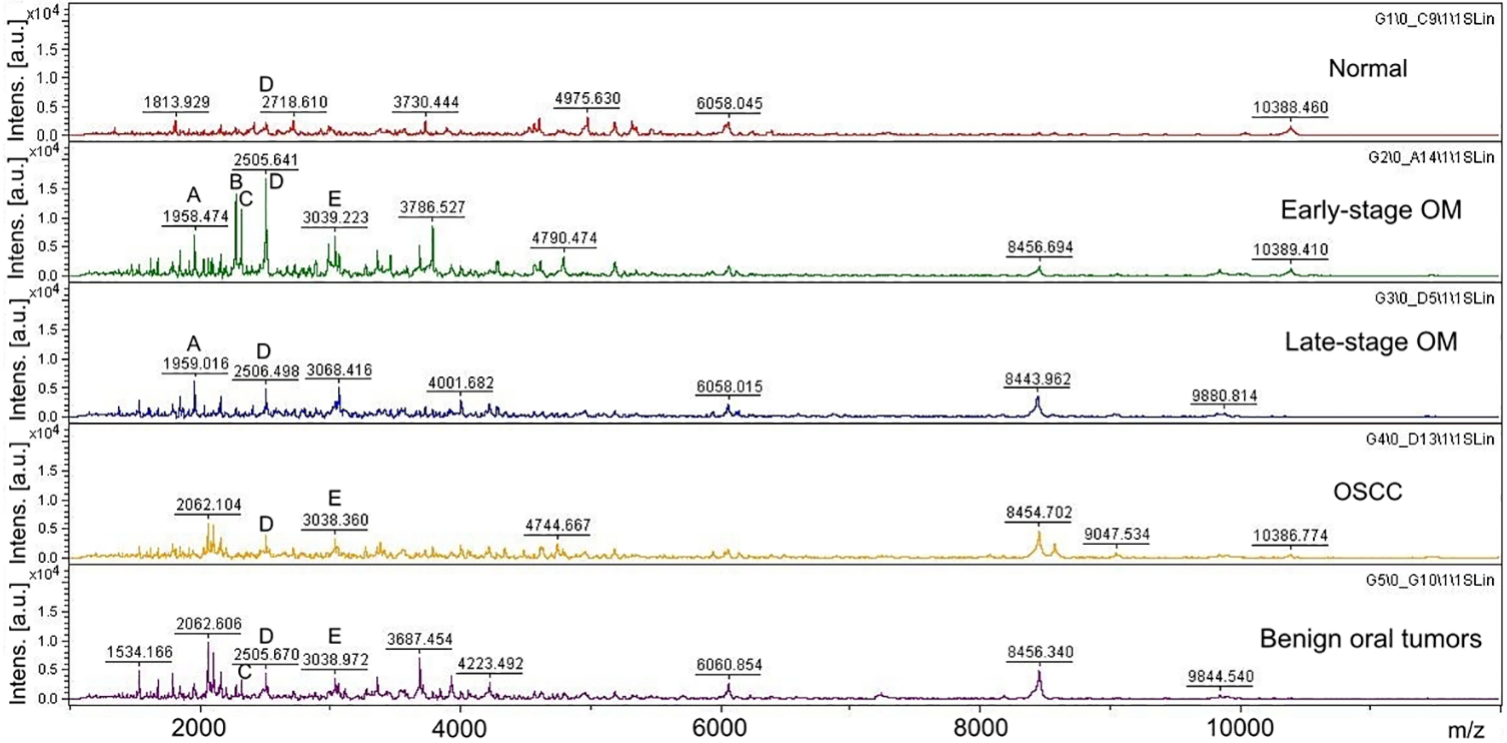
Peptide mass fingerprint of early-stage oral melanoma (OM), late-stage OM, oral squamous cell carcinoma (OSCC), benign tumors and normal gingiva tissues in the range of 1,000–10,500 Da with identified proteins of each mass spectrum. A: SCN10A (1,958 Da); B: SACS (2,274 Da); C: NOTCH1 (2,316 Da); D: GRIN3A (2,505 Da); E: LARS (3,039 Da).

**Fig 2:**
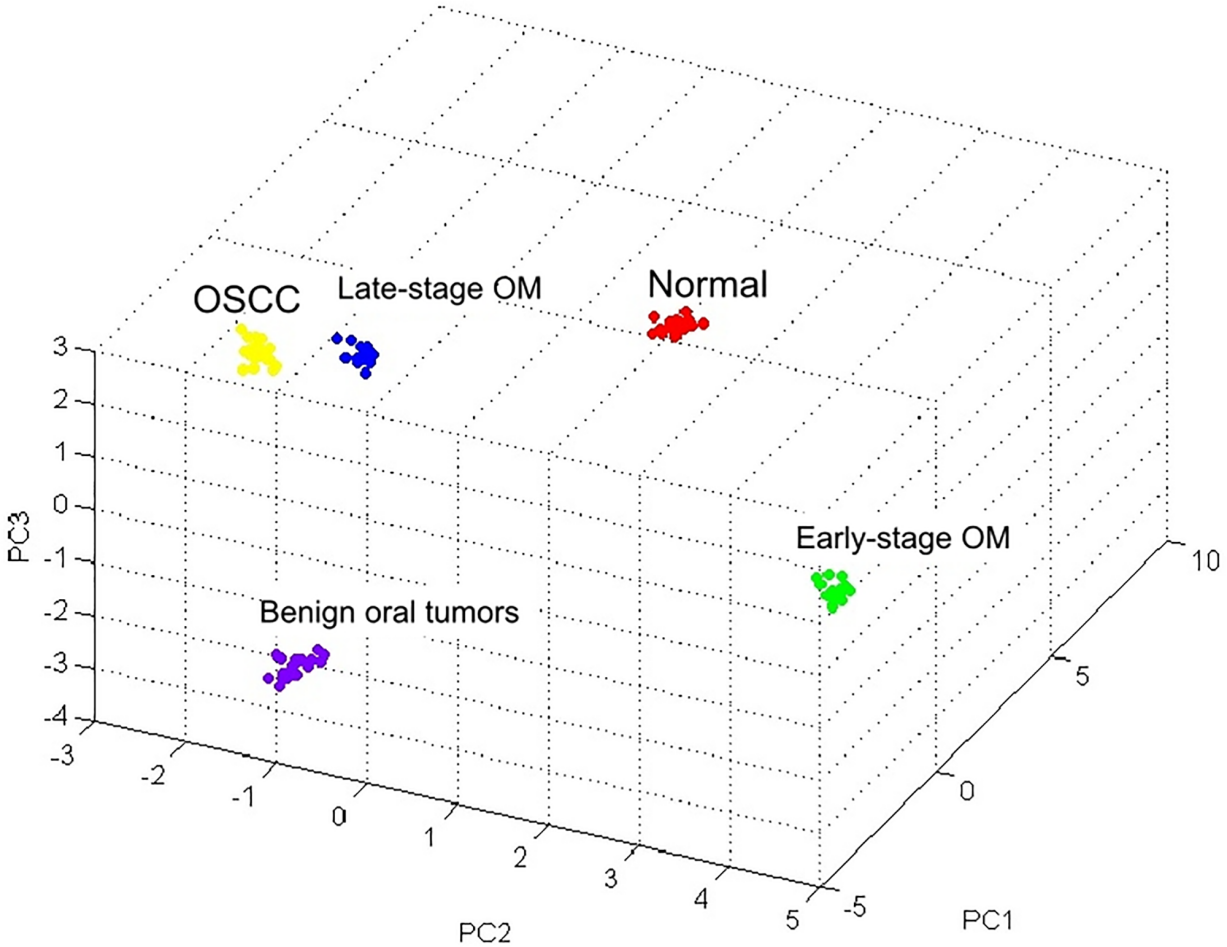
The 3-dimensional principal component analysis (3D PCA) scatterplot of normal gingiva tissues, early-stage oral melanoma (OM), late-stage OM, oral squamous cell carcinoma (OSCC) and benign tumors.

Different PMFs of normal gingiva tissues, early-stage OM, late-stage OM, OSCC and benign tumors were detected in the range 1,000–10,500 Da (Fig 1). A number of unique peaks distinguishing each group were observed in addition to some common peaks among different groups as demonstrated in Table 2. The 3D view of the plot analysis of PCA scores exhibited a discrete cluster of each sample group that was clearly distinguished from the others, indicating similarity within a group (Fig 2). The MALDI-TOF MS results had an accurate outcome with the 95% confidence interval. The cross validation, calculated by Kruskall-Wallis test, in the normal controls, early-stage OM, late-stage OM, OSCC and benign oral tumors was 100%, 100%, 94.87%, 100% and 100%, respectively, and the recognition capability, calculated by Kruskall-Wallis test, in the normal controls, early-stage OM, late-stage OM, OSCC and benign oral tumors was all 100%, indicating that the results were of high reliability.

**Table 2:** Comparison of peaks in MALDI-TOF analysis of normal controls, benign tumors, early- and late-stage oral melanoma (OM) and oral squamous cell carcinoma (OSCC).

Sample groups	Unique markers (m/z)	Common markers between two groups (m/z)	Common markers among three groups (m/z)	Common markers among five groups (m/z)
**Normal**	–	–	–	2505*
**Early OM**	2274*, 3786	1846, 1958*, 2316*, 3687, 8457	3039*	2505*
**Late OM**	–	1846, 1958*	–	2505*
**OSCC**	8577	2062	3039*	2505*
**Benign tumors**	1534, 4223	2062, 2316*, 3687, 8457	3039*	2505*

Asterisks (*) represent candidate peptide masses selected by ClinProTools software for further analysis by LC MS/MS.

**Fig 3:**
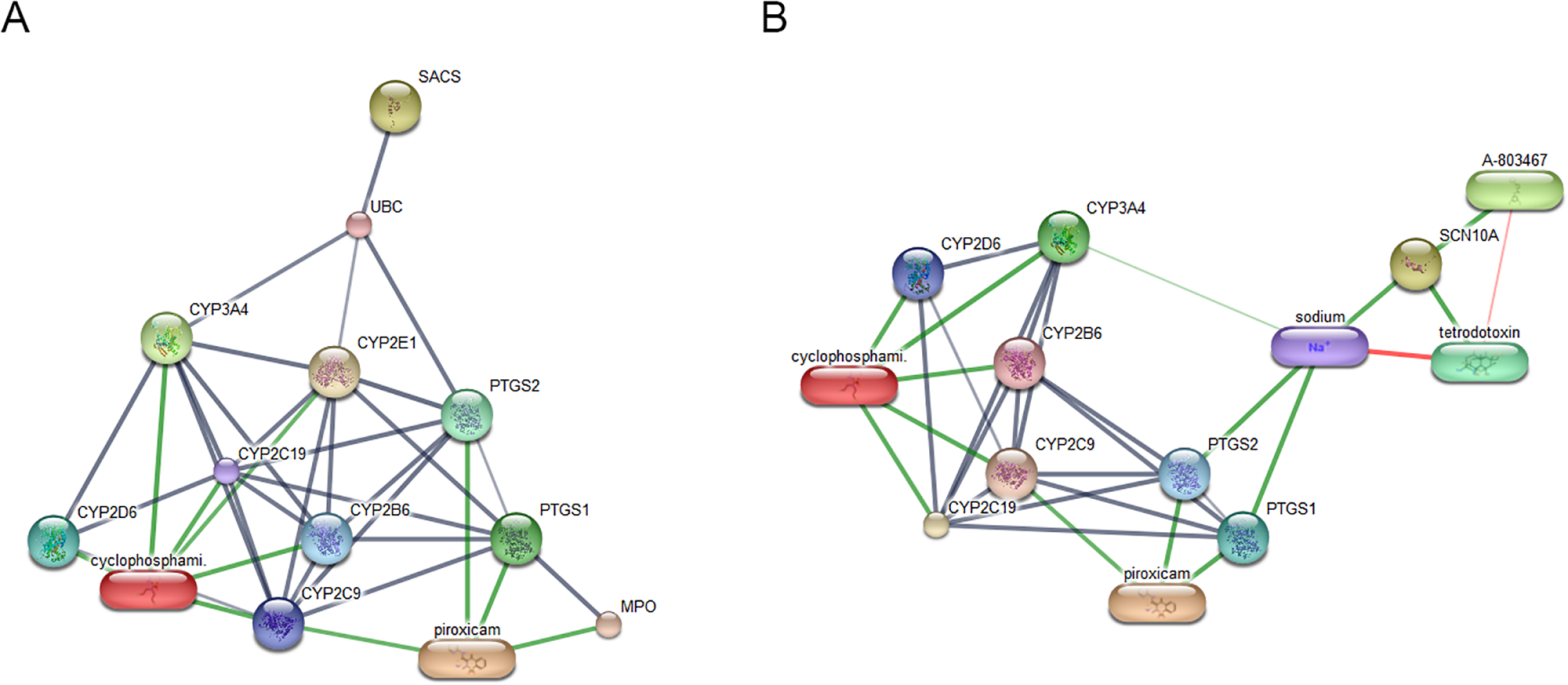
The involvement of sacsin (SACS) (A) and sodium channel protein type 10 subunit alpha (SCN10A) (B) in networks of protein-chemotherapy drug interactions, cyclophosphamide and piroxicam, analyzed by the Stitch program version 5.0. Red circles: SACS and SCN10A. Abbreviations: cytochrome P450 family 3 subfamily A member 4 (CYP3A4), cytochrome P450 family 2 subfamily B member 6 (CYP2B6), cytochrome P450 family 2 subfamily C member 9 (CYP2C9), cytochrome P450 family 2 subfamily C member 19 (CYP2C19), cytochrome P450 family 2 subfamily D member 6 (CYP2D6), cytochrome P450 family 2 subfamily E Member 1 (CYP2E1), myeloperoxidase (MPO), prostaglandin-endoperoxide synthase 1 (PTGS1), prostaglandin-endoperoxide synthase 2 (PTGS2) and ubiquitin C (UBC).

Proteins expressed high signal intensities either individually found in each sample group or commonly found in normal and benign tumor, early- and late-stage OM, and only cancerous groups were analyzed by LC-MS/MS. In addition, protein lists were submitted to the PANTHER classification system. The results showed the association of these proteins with the molecular function, biological process and cellular component (Table 3). Proteins function through interaction with other proteins or molecules. Networks of protein–protein and protein–chemotherapy drug interactions were analyzed by the Stitch program, version 5.0. Edge confidence scores were used to represent the strength of the protein–protein interactions at the functional level. Pathways with high edge confidence scores (>0.700) were demonstrated as thick lines. The correlation of sacsin (SACS) and sodium channel protein type 10 subunit alpha (SCN10A) with cyclophosphamide and piroxicam, common drugs used in metronomic therapy, was observed (Fig 3).

**Table 3:** Overexpressed proteins based on biological process involvement and protein score.

Database	Protein name	Protein ID score	Peptides	Biological process	Subcellular distribution
**gi|545538198**	Sodium voltage-gated channel alpha subunit 10 (SCN10A)	14.8	AFEAMLQIGNIVFTVFF	Cell action potential	Cell membrane
**gi|545542017**	Sacsin molecular chaperone (SACS)	20.3	VLSDQAYSELLGLELLPLQNG	Chaperone binding	Mitochondria and nucleus
**gi|545513541**	Notch 1 (NOTCH1)	21.3	VLGTGSGSTSGSGGGAVNFTMGGATAL	Cell differentiation	Endoplasmic reticulum, Nucleus and cell membrane
**gi|545517787**	Glutamate ionotropic receptor NMDA type subunit 3A (GRIN3A)	23.1	VTVSILTMNNWYNFSLLLCQE	Calcium ion transport	Cell membrane
**gi|545490543**	Leucine-tRNA synthetase (LARS)	21.7	QLKQEFEFWYPVDLRVSGKDLVPNH	Cellular response to leucine	Cytoplasm

Data were achieved by MALDI-TOF MS and LC-MS/MS.

**Fig 4:**
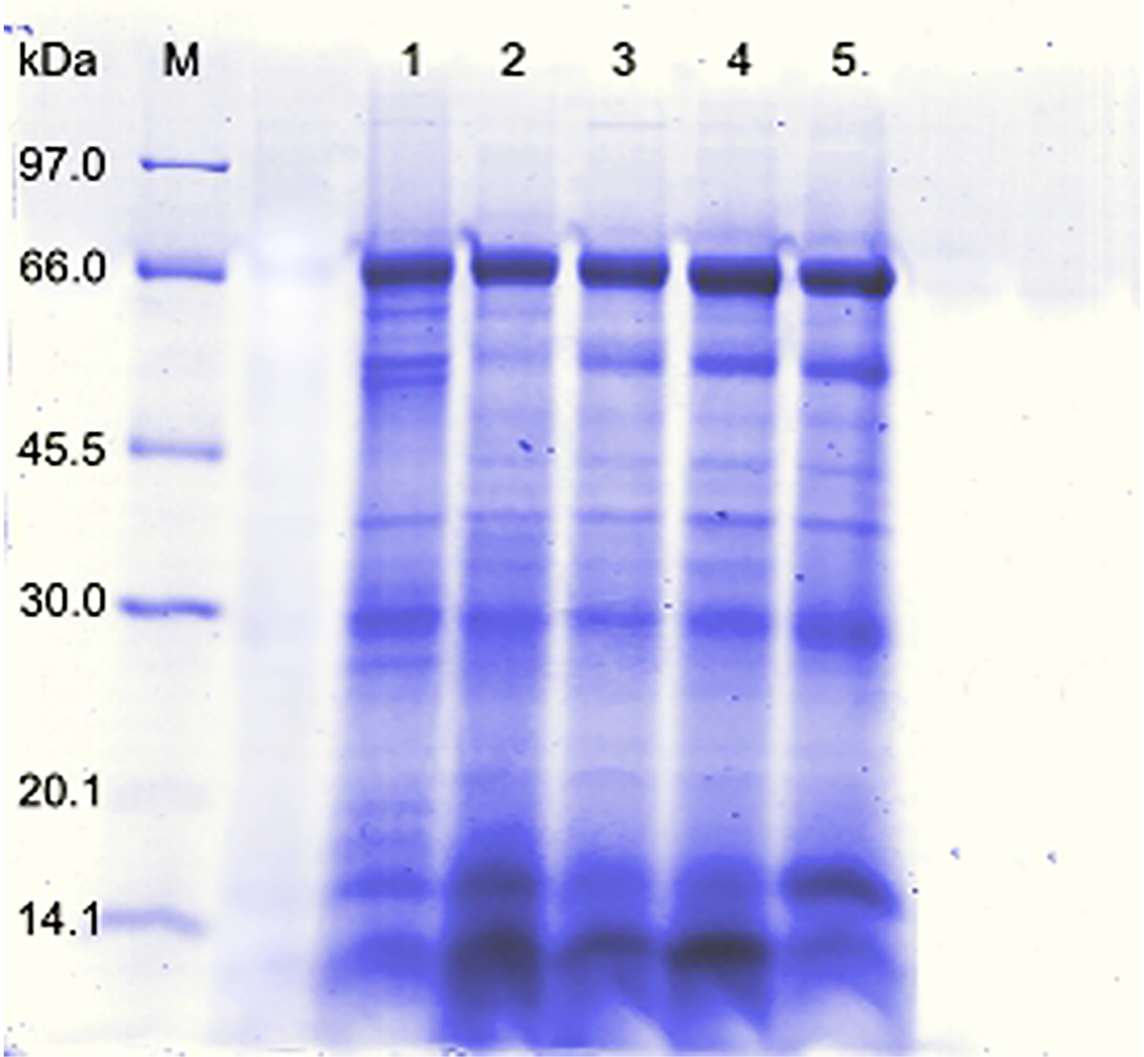
Analysis of denatured protein on 12% SDS-PAGE. lane 1: crude extract of normal gingiva tissues; lane 2: crude extract of early-stage oral melanoma (OM); lane 3: crude extract of the late-stage OM; lane 4: oral squamous cell carcinoma; lane 5: benign tumors; lane M: protein molecular weight marker.

**Fig 5:**
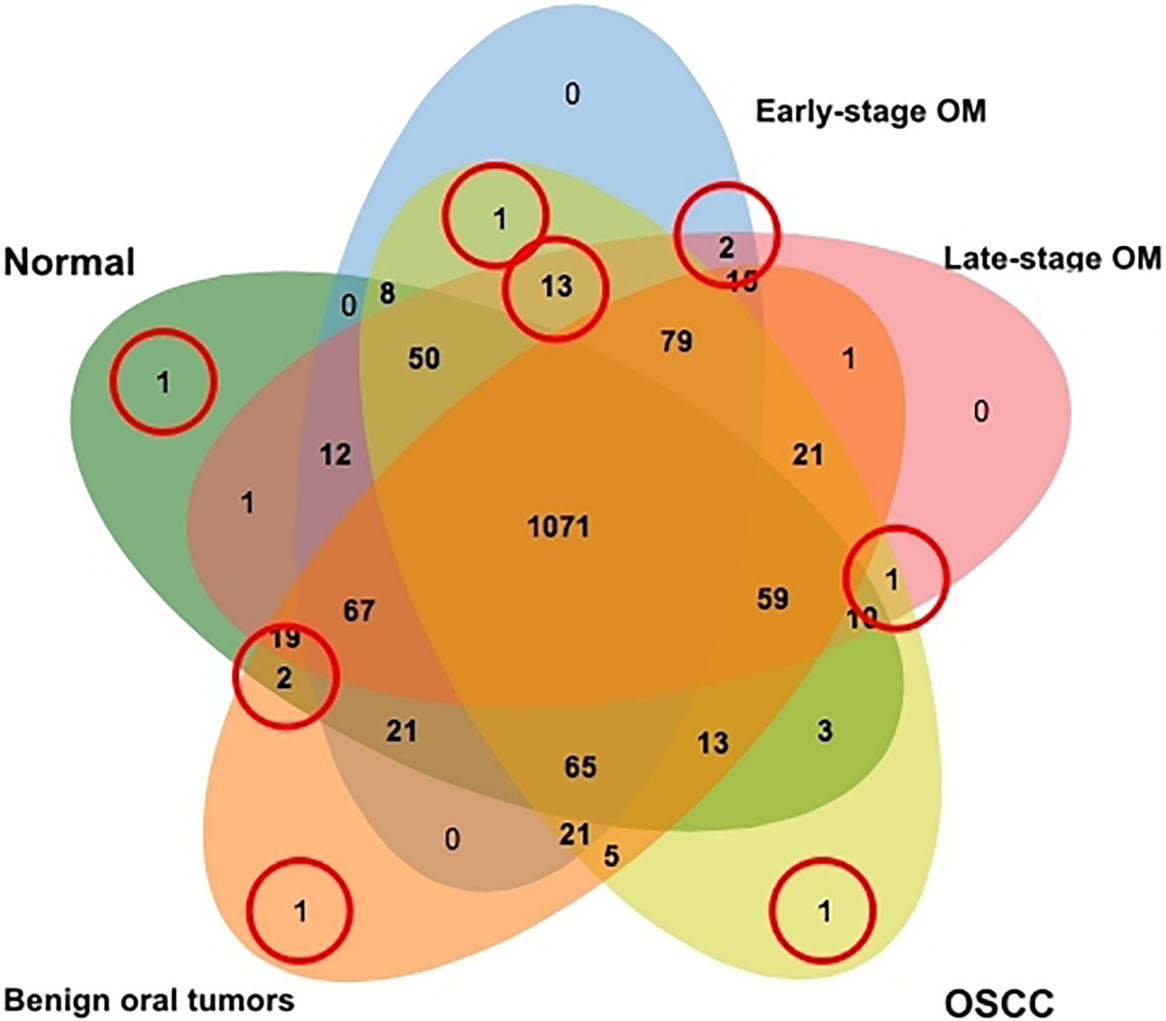
Venn diagram of proteins differentially expressed in early-stage oral melanoma (OM), late-stage OM, oral squamous cell carcinoma (OSCC), benign oral tumors (Benign) and normal gingiva tissues (NR). Circles indicate overexpressed proteins either individually found in each sample group or commonly found in normal and benign tumor, early- and late-stage OM, and only cancerous groups.

**Fig 6:**
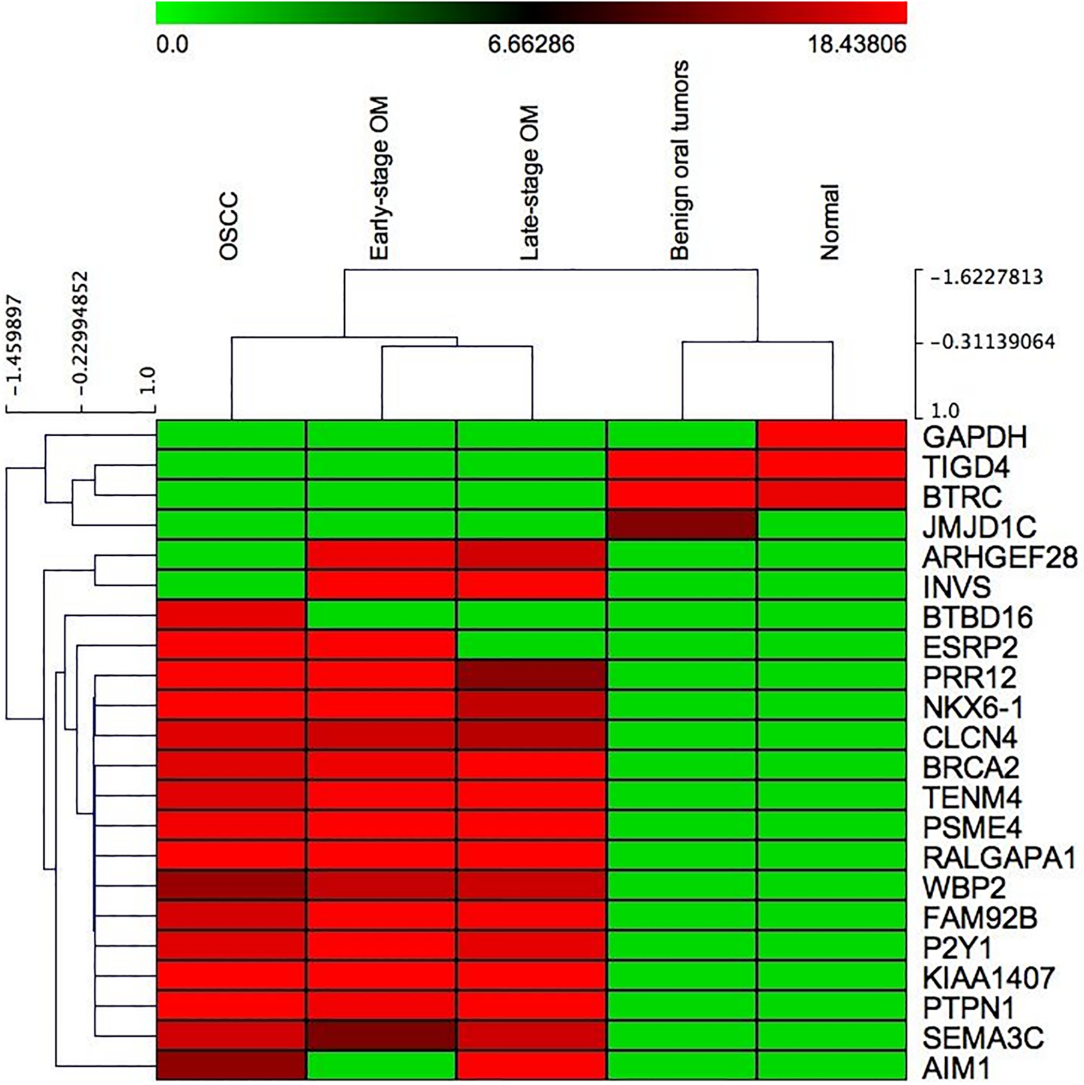
Hierarchical clustering and heat map of differentially expressed proteins in canine oral squamous cell carcinoma (OSCC), early-stage oral melanoma (OM), late-stage OM, benign oral tumors and normal gingiva tissues (Normal). Green color indicates downregulated proteins and red color indicates upregulated proteins among different sample groups.

For the GeLC-MS/MS analysis, the pooled protein samples from normal, early-stage OM, late-stage OM, OSCC and benign oral tumors were separated according to their molecular weight by 12% SDS-PAGE. The different patterns of proteins among 5 groups are shown in Fig 4. A total of 1,572 proteins were identified. The distribution of the unique and overlapped proteins among sample classes was depicted using Venn diagrams (Fig 5). Proteins expressed high signal intensities either individually found in each sample group or commonly found in normal and benign tumor, early- and late-stage OM, and only cancerous groups were indicated. In addition, the PANTHER classification system was used to reveal the association of these proteins with the molecular function, biological process and cellular component (Table 4 and S1 Table). In the S1 Table, the relative expression levels of distinct proteins in normal controls, benign tumors, early- and late-stage oral melanoma (OM) and oral squamous cell carcinoma (OSCC) were shown as log2 intensities [48]. Protein scores (ID scores) were derived from ion scores as a non-probabilistic ranking protein hits and obtained as the sum of peptide scores. The score threshold was set at p<0.05 by Mascot algorithm. Protein name was used instead of ID best hit. Proteins with unique signal intensities in each group and also associated with carcinogenesis and chemotherapy analyzed by the Stitch program [46], were selected as potential candidate biomarkers of the diseases. The hierarchical clustering analysis was performed to study the similarities in protein expression profile. Early-, late-stage OM and OSCC were closely clustered, reflecting a similar protein expression profile in these groups whereas benign tumors and normal controls formed a distinct sub-cluster (Fig 6).

**Table 4:** Overexpressed proteins in normal controls, benign tumors, early- and late-stage oral melanoma (OM) and oral squamous cell carcinoma (OSCC) based on biological process involvement and protein score.

Database	Protein name	Protein ID score	Peptides	Biological process	Subcellular distribution
**Normal control**:					
**gi|545559938**	Glyceraldehyde-3-phosphate dehydrogenase (GAPDH)	11.55	VQPNEAVYTKMMTK	Metabolic process	Cytoplasm
**Benign tumors**:					
**gi|545495008**	Jumonji domain containing 1C (JMJD1C)	3.82	QPKPTYK	Transcription	Nucleus
**Normal control and benign tumors**:					
**gi|545524870**	Tigger transposable element derived 4 (TIGD4)	8.55	MAEASEDASALPMTVK	DNA binding	Nucleus
**gi|545547706**	Beta-transducin repeat containing E3 ubiquitin protein ligase (BTRC)	3.36	TLNGHKR	Protein modification	Cytoplasm, nucleus
**Early- and late-stage OM**:					
**gi|545516425**	Inversin (INVS)	7.89	WNRECLALLLQVWR	Organism development	Cytoplasm, cell membrane and nucleus
**gi|545490929**	Rho guanine nucleotide exchange factor 28 (ARHGEF28)	4.39	EVANEER	Apoptotic process and cell adhesion	Cytoplasm, cell membrane
**OSCC**:					
**gi|545548178**	BTB domain-containing 16 (BTBD16)	9.70	VAFATALK	apoptotic process and cellular protein modification process	Nucleus
**Early- and late-stage OM, and OSCC**:					
**gi|545541064**	Protein tyrosine phosphatase non-receptor type 1 (PTPN1)	23.64	KVLLEMR	Cellular protein modification process	Cytoplasm
**gi|545536197**	Teneurin 4 (TENM4)	16.12	FFVMETIIMR	Organism development	Cytoplasm, cell membrane and nucleus
**gi|345786077**	Proline rich 12 (PRR12)	12.83	LEPLKPLK	Neuromuscular process controlling posture	Cell membrane, cell junction
**gi|545552897**	Coiled-coil domain-containing 191 (KIAA1407)	10.02	QEENSPK	–	Nucleus
**gi|74001795**	NK6 Homeobox 1(NKX6-1)	9.13	QDSETERLK	Organism development and transcription	Nucleus
**gi|58801256**	BRCA2, DNA repair associated (BRCA2)	8.13	LAAMEFAFPKEFANR	Biosynthetic process and transcription	Nucleus
**gi|545507136**	Ral GTPase-activating protein catalytic alpha 1 subunit (RALGAPA1)	5.82	QHTEEKEFVEK	catabolic process and intracellular signal transduction	Cytoplasm
**gi|73981987**	Semaphorin 3C (SEMA3C)	4.24	QIHSMIAR	Locomotion	Extracellular space
**gi|74006437**	Chloride voltage-gated channel 4(CLCN4)	3.86	ELILAIK	Anion transportation	Endosome membrane and endoplasmic reticulum membrane
**gi|545499696**	Family with sequence similarity 92 member B (FAM92B)	3.08	LEPLKPLK	Cilium biogenesis/degradation	Cytoplasm
**gi|545509413**	WW domain binding protein 2 (WBP2)	2.62	VIFLSKGK	Transcription	Nuclear chromatin
**gi|52788591**	Purinergic receptor P2Y1 variant 2 (P2Y1)	2.35	ALIYKDLDDSPLR	phospholipase C-activating G-protein coupled receptor signaling pathway	Cell membrane
**gi|545515591**	Proteasome activator subunit 4 (PSME4)	1.06	DPGSVGDTIPSAELVKR	cellular response to DNA damage stimulus	Cytoplasm, nucleus
**Early-stage OM and OSCC**:					
**gi|545500178**	Epithelial splicing regulatory protein 2 (ESRP2)	2.27	YVEVVPCSTEEMSR	mRNA splicing, via spliceosome and positive regulation of epithelial cell proliferation	Nucleus
**Late-stage OM and OSCC**:					
**gi|545520349**	Absent in melanoma 1 protein (AIM1)	7.10	QFLLSPAEVPNWYEFSGCR	carbohydrate binding	Nucleus

Data were achieved by GeLC-MS/MS.

**Fig 7:**
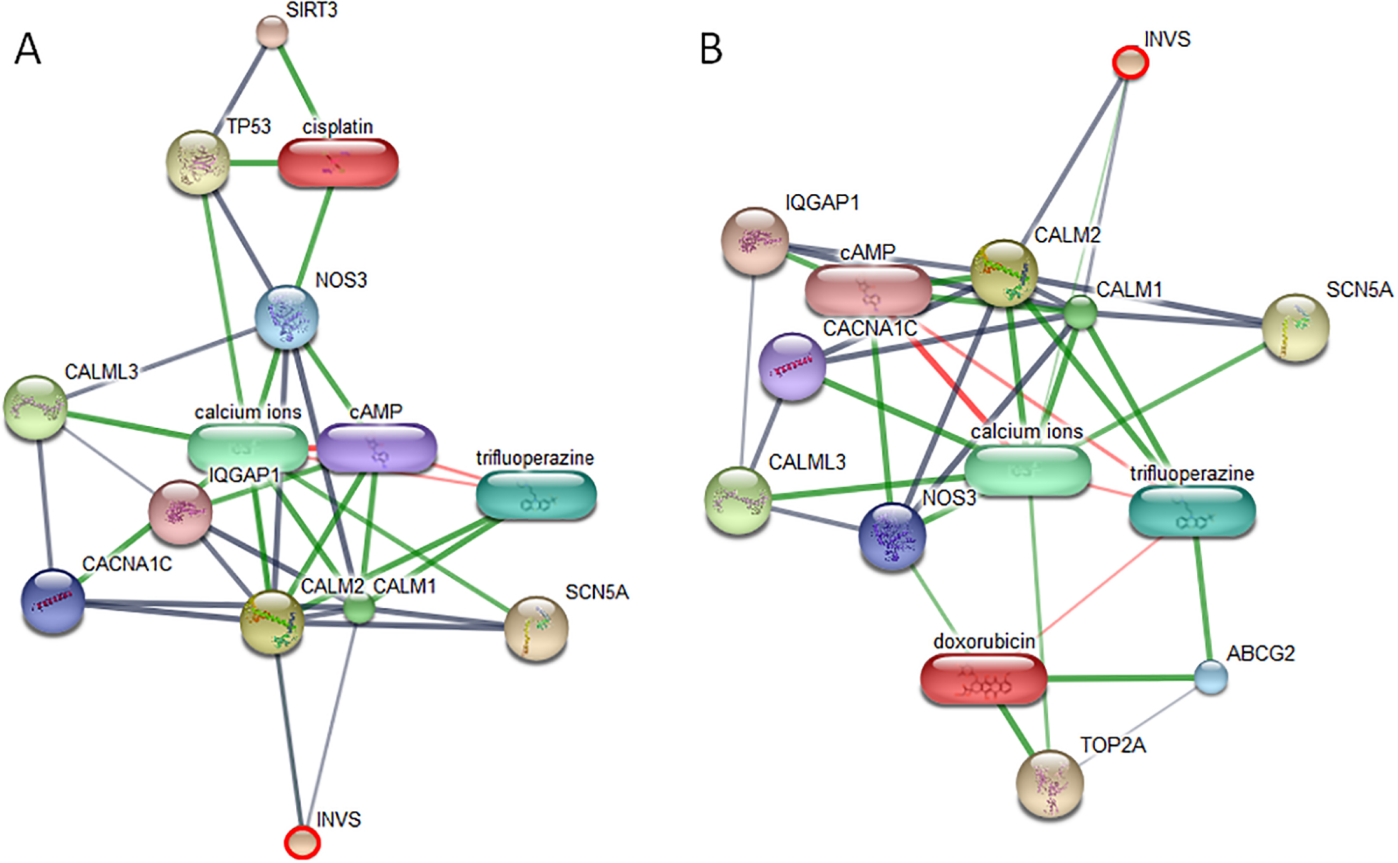
The involvement of inversin (INVS) in networks of protein-chemotherapy drug interactions, cisplatin (A) and doxorubicin (B), analyzed by the Stitch program version 5.0. Red circles: INVS. Abbreviations: ATP-binding cassette sub-family G member 2 (ABCG2), voltage-dependent L-type calcium channel subunit alpha-1C (CACNA1C), calmodulin 1 (CALM1), calmodulin 2 (CALM2), calmodulin-like 3 (CALML3), ras GTPase-activating-like protein IQGAP1 (IQGAP1), nitric oxide synthase 3 (NOS3), sodium channel protein type 5 subunit alpha (SCN5A), sirtuin 3 (SIRT3), topoisomerase (DNA) II alpha (TOP2A) and tumor protein p53 (TP53).

**Fig 8:**
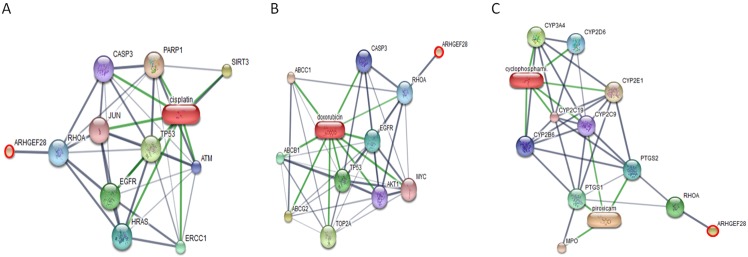
The involvement of rho guanine nucleotide exchange factor 28 (ARHGEF28) in networks of protein-chemotherapy drug interactions, cisplatin (A) and doxorubicin (B), analyzed by the Stitch program version 5.0. Red circles: ARHGEF28. Abbreviations: multidrug resistance protein 1 (ABCB1), multidrug resistance-associated protein 1 (ABCC1), ATP-binding cassette sub-family G member 2 (ABCG2), RAC-alpha serine/threonine-protein kinase (AKT1), serine-protein kinase ATM (ATM), caspase 3 (CASP3), cytochrome P450 3A4 (CYP3A4), cytochrome P450 2B6 (CYP2B6), cytochrome P450 2C9 (CYP2C9), cytochrome P450 2C19 (CYP2C19), cytochrome P450 2D6 (CYP2D6), cytochrome P450 2E1 (CYP2E1), epidermal growth factor receptor (EGFR), excision repair cross-complementing rodent repair deficiency, complementation group 1 (ERCC1), Harvey rat sarcoma viral oncogene homolog (HRAS), jun proto-oncogene (JUN), myeloperoxidase (MPO), myc proto-oncogene protein (MYC), poly (ADP-ribose) polymerase 1 (PARP1), prostaglandin G/H synthase 1 (PTGS1), prostaglandin G/H synthase 2 (PTGS2), ras homolog family member A (RHOA), sirtuin 3 (SIRT3), topoisomerase (DNA) II alpha (TOP2A) and tumor protein p53 (TP53).

**Fig 9:**
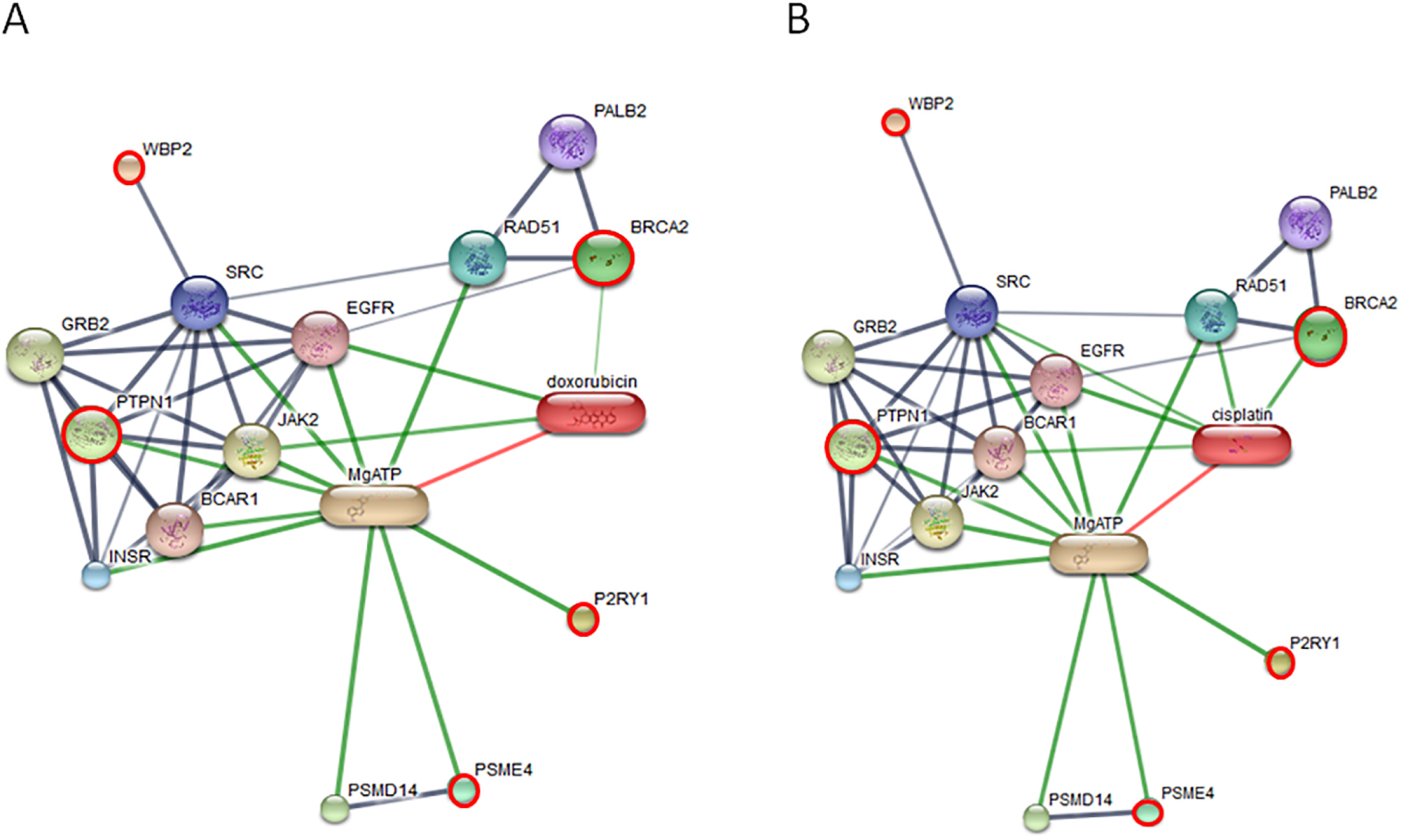
The involvement of tyrosine-protein phosphatase non-receptor type 1 isoform X3 (PTPN1), BRCA2, WW domain-binding protein 2 isoform X4 (WBP2), purinergic receptor P2Y1 variant 2 (P2RY1) and proteasome activator complex subunit 4 isoform X3 (PSME4) in networks of protein-chemotherapy drug interactions, cisplatin (A) and doxorubicin (B), analyzed by the Stitch program version 5.0. Red circles: PTPN1, BRCA2, P2RY1, PSME4 and PTPN1. Abbreviations: anti-estrogen resistance protein 1 (BCAR1), growth factor receptor-bound protein 2 (GRB2), epidermal growth factor receptor (EGFR), insulin receptor (INSR), tyrosine-protein kinase JAK2 (JAK2), partner and localizer of BRCA2 (PALB2), 26S proteasome non-ATPase regulatory subunit 14 (PSMD14), DNA repair protein RAD51 homolog 1 (RAD51) and proto-oncogene tyrosine-protein kinase Src (SRC).

Networks of protein–protein and protein–chemotherapy drug interactions were analyzed by the Stitch program, version 5.0. The involvement of INVS, ARHGEF28, PTPN1, BRCA2, WBP2, P2RY1 and PSME4 in networks of protein-chemotherapy drug interactions, cisplatin and doxorubicin, was analyzed (Figs 7–9). Except ARHGEF28, no or very faint correlation of target proteins with cyclophosphamide/piroxicam, common drugs used in metronomic therapy, was observed. And no correlation of all drugs and BTBD16 was exhibited as well as the correlation of the drugs and a number of proteins in an all cancer group, including, teneurin transmembrane protein 4 (TENM4), coiled-coil domain containing 191 (KIAA1407), NK6 homeobox 1 (NKX6-1), ral GTPase-activating protein subunit alpha-1 (RALGAPA1), semaphorin 3C (SEMA3C), chloride voltage-gated channel 4 (CLCN4) and family with sequence similarity 92 member B (FAM92B). However, the reason may be due to the fact that the connection of those proteins and chemotherapy drugs has never been discovered.

## Discussion

This study initially used the top-down MS-based approach, MALDI-TOF MS, to exhibit PCA plots and PMFs of OM, OSCC, BN and normal control groups. Identification of proteins underlying discriminatory peaks was performed by LC-MS/MS. The bottom-up GeLC-MS/MS approach was used to identify protein markers in each group. Different PMFs and a number of unique peaks as well as a discrete cluster from the PCA analysis were exhibited in each sample group. Hence, MALDI-TOF MS can possibly be used as rapid and reliable diagnostic tools for detection of canine oral tumors. MALDI analysis has been used for the diagnosis of human head and neck squamous cell carcinoma and human OSCC from oral brush biopsy and oral fluid [49–51]. Since we could distinguish early- and late-stage OM by MALDI-TOF MS, the clinical application of this technique in early detection of the disease, which implies the early treatment, the better quality of life of the patients and probably higher survival rate, would be possible. The technique is suitable to fulfill the diagnosis since the conventional histological analysis had lengthy processes and required experts in veterinary pathology to interpret results where discordance usually exists among specialists. In fact, histology-directed analysis of tissue sections which combines tissue section and MALDI-TOF MS to target specific cells are currently interested [52]. However, more number of clinical samples is required to set databanks of PMFs and PCA plots.

This study also revealed the candidate proteins from MALDI mass spectra. SACS is a co-chaperone of heat shock protein (HSP)70. It is required for proper folding and function of HSP70 chaperone proteins [53]. HSP70 was target therapy for cancers since a number of cancers overexpress HSP70 family members [54, 55]. However, the association of SACS and HSP in canine oral cancer has not been reported. SCN10A, exclusively identified in OM groups, is one of the voltage-gated sodium channels (VGSC) proteins. The alteration of the Na^+^ ions at the cell membrane is important for cell proliferation, especially cancer cells [56]. Enhanced protein expression in a VGSC group, such as SCN5A in human breast cancer and SCN4A in prostate cancer, was reported to be associated with cancer invasiveness [57, 58]. Thus, the SCN10A might affect the canine OM progression. Predicted interaction between SACS or SCN10A proteins and chemotherapy drugs, cyclophosphamide/piroxicam, was exhibited in the present study via several cytochrome P450 (CYP) proteins which plays an important role in cancer development and response to therapy [59–62].

For Notch1, the protein in the Notch signaling pathway, it was also involved in tumor progression [63]. Notch1 associated with epithelial-mesenchymal transition (EMT) in prostate cancer. Inhibition of Notch1 decreased the proliferation of melanoma cell line [64, 65]. In the present study, Notch 1 expression was found in the early-stage OM and benign oral tumors, the relationship of Notch 1 expression and early detection of OM should be further investigated. LARS, another candidate found in several tumor groups, was previously discovered as the anticancer target due to the inhibition of nutritional pathway of cancer cells [66]. Hence, the inhibition of LARS in oral cancers and tumors in dogs should be further investigated for the possibility to be developed as targeted therapy. However, all of the candidate biomarkers from this study should be confirmed by other techniques such as western blotting.

With GeLC-MS/MS, a number of proteins were shown to be increasingly expressed in canine oral tumors whereas GAPDH was remarkably observed only in normal controls, indicating it might not be a suitable housekeeping gene/protein for gene expression study in oral cavity in dogs. As GAPDH functions to generate NADH from NAD^+^ in glycolysis pathway. In general, increased GAPDH expression was shown in cancer owing to the NAD^+^ supply by lactate dehydrogenase in anaerobic glycolysis [67]. However, in our case, lack of GAPDH in tumor cells may be due to depleted NAD^+^, probably indicating aberrant anaerobic glycolysis in canine oral tumors. JMJD1C isoform X5 was found to be increased in the benign tumors. JMJD1C plays an important role in the histone demethylation as an epigenetic regulation. Decreased expression of the JMJD1C variant, s-JMJD1C, was observed in breast cancer whereas in normal breast tissues the expression was significantly increased, suggesting its functions in tumor suppression [68]. In our study, JMJD1C was solely found in benign tumors, not in any cancers or normal tissues, probably indicating a potential role of JMJD1C as a biomarker for benign tumors of the oral cavity in dogs.

In both early- and late-stage OM, INVS isoform X6 was found to be overexpressed. As INVS has two IQ calmodulin (CALM) domains, we added keywords: inversin, calmodulin 1, calmodulin 2, calmodulin 3 and cisplatin or doxorubicin or cyclophosphamide/piroxicam, to the Stitch program [69]. A link between INVS, a unique protein in an OM group, and cisplatin or doxorubicin via CALM2, calcium ions, and tumor protein p53 (TP53) or nitric oxide synthase 3 (NOS3) was proposed. CALM is the calcium-binding protein that regulates cellular proliferation. As CALM is a multi-phase protein, paradoxical roles of CALM have been reported. CALM inhibition could induce proliferative arrest and apoptosis mediated by activation of a tumor suppressor, TP53, and restore resistant cell sensitivity to chemotherapy drugs such as doxorubicin [70, 71], whereas CALM and calcium played an important role in cisplatin-induced tumoricidal activity of peritoneal macrophages in mouse [72]. The low levels of NOS, a CALM -dependent enzyme, also participated in the induction of cell proliferation, as NOS and CALM inhibitors could inhibit this process [70]. In OSCC, BTBD16 was markedly expressed. The BTB/POZ domain is a common structural domain in several proteins. BTBD7 enhanced hepatocellular carcinoma (HCC) angiogenesis and metastasis, hence, promoting HCC progression [73]. Zinc finger and BTB domain-containing protein 3 played an important role in the in human melanoma, lung carcinoma, and breast carcinoma cell growth via the reactive oxygen species (ROS) detoxification pathway [74]. The rho guanine nucleotide exchange factor 28 (ArhGEF28 or p190RhoGEF or Rgnef), a member of the Dbl family of RhoGEFs which promote the active GTP-bound state of Rho GTPases, was found to be expressed in early- and late-stage OM [75, 76]. Elevated ArhGEF28 expression promotes colorectal carcinoma invasion and tumor progression via interaction with focal adhesion kinase [77, 78]. ArhGEF28 interacts with RHOA which was found to be overexpressed in several cancers such as prostate cancer [79], gastric cancer [80] and chronic myeloid leukemia [81]. Decreased RhoA protein expression by RhoA small interfering RNA was associated with the increased sensitivity to doxorubicin in human colon cancer cell line [82].

A number of expressed proteins in the cancerous group (OM in all stages and OSCC) were found in protein–chemotherapy drug interactions (cisplatin and doxorubicin) including PTPN1, BRCA2, WBP2, P2Y1 and PSME4 (Figs 7–9). PTPN1 and BRCA2 expression was possibly a negative feedback of the tumorigenesis. PTPN13 inhibited hepatocellular carcinoma through inactivation of the epidermal growth factor receptor (EGFR)/extracellular signal-regulated kinase (ERK) signaling pathway [83]. The elevated expression of epidermal growth factor (EGF), EGFR, Janus kinase (JAK) and proto-oncogene tyrosine-protein kinase Src (Src) is noted in human oral cancer [84]. Activation of EGFR/Erk1/2 and JAK could enhance invasiveness of cisplatin-resistant ovarian cancer cells in vitro, and the inhibition of both EGFR and JAK appeared to be an efficient approach to treat human ovarian cancer [85, 86]. Increased EGFR expression was related to doxorubicin resistance in lung cancer cells [87]. BRCA2 is a tumor suppressor that prevent cells from growing and dividing too rapidly [88]. Mutation of BRCA2 in breast and ovarian cancers compromises DNA homologous repair and then leads to BRCA-associated tumors sensitive to cisplatin, which causes DNA breaks and requires a repair process [89]. However, secondary mutation of BRCA2 can elicit cisplatin resistance in ovarian carcinomas [90].

WBP2 is a tyrosine kinase substrate. The enzyme can phosphorylates targets and induce tumorigenesis [91]. In human breast cancer, phosphorylation of WBP2 at Tyr192 and Tyr231 was regulated by c-Src and c-Yes kinases and was stimulated by EGF. WBP2 tyrosine phosphorylation could enhance the transcription of estrogen receptor α, which induced the angiogenesis of breast cancer [92, 93]. The P2Y1 receptor belongs to a family of G protein-coupled receptor. In human prostate cancer and melanoma, the P2Y1 receptor induces cell apoptosis and/or inhibits cell proliferation and is a putative target for cancer therapy [94, 95]. The role of P2Y1 receptor in canine oral cancer requires further investigation. Proteasomes, a multisubunit protein complex, function to destroyed unnecessary or damaged proteins. Enriched proteasome genes, including PSME4 and PSMD14, was exhibited in doxorubicin-derived resistant ovarian cancer cell line, suggesting that this proteasome pathway may be involved in the development of resistance to doxorubicin [96]. As various drugs have been used to treat dogs with oral malignant tumors following surgical excision, in the present study we demonstrated relationships between chemotherapy drugs and disease-perturbed proteins, which might help veterinarians choose drugs of choice and treatment plan. Further studies are required for the effects of drugs on the protein biomarkers of the diseases.

In our study, we obtained different expressed proteins from MALDI-TOF MS combined with LC-MS/MS and from GeLC-MS/MS. The plausible explanations included different types of ionization techniques, the sample preparation steps and the statistical analysis. The reason why proteins from GeLC MS/MS did not appear in results from MALDI-TOF MS combined with LC-MS/MS might be associated with the ZipTip cleanup in the sample preparation steps for MALDI-TOF MS. Since peptide masses were ZipTip purified and eluted through the 2% series of ACN, the amount of each peptide mass was lessen and might not be able to be detected by LC-MS/MS technique whereas the GeLC-MS/MS could identify proteins from intact peptides. On the other hand, the reason why proteins from MALDI-TOF MS combined with LC-MS/MS did not appear in results from GeLC MS/MS was probably due to T-test/ANOVA statistics (p<0.05) performed to select significant proteins in data processing of GeLC-MS/MS. Therefore, the candidate proteins from MALDI-TOF MS combined with LC-MS/MS might not be present in GeLC-MS/MS analysis. A limitation of the current study was probably the inability to specify whether the peptides were from the tumor cells, stroma or elsewhere (e.g., interstitial fluid) because in our study we did not perform primary culture or using the flow cytometer to separate solely tumor cells for protein extraction.

## Conclusions

The present study revealed the distinct cluster of each sample group, unique **PMFs** and protein identification in OM, OSCC, BN and normal control subjects, using MALDI-TOF MS combined with LC-MS/MS, and also identified potential protein candidates associated with the diseases, using GeLC-MS/MS. The network connections between these proteins and the chemotherapy drugs, cisplatin, doxorubicin, cyclophosphamide and piroxicam, were also demonstrated. For future work, protein–protein interaction in the diseases should be confirmed by high-throughput approaches, such as yeast two-hybrid screening and affinity purification coupled to mass spectrometry [97, 98].

## Supporting information

S1 Fig Histopathological features of melanotic melanoma (A), amelanotic melanoma (B), well differentiated squamous cell carcinoma (C), poorly differentiated squamous cell carcinoma (D), epulis (E) and ameloblastoma (F). Bar, 50 mm, Inset: Bar, 20 mm.
(TIF)

S1 Table The relative expression levels of distinct proteins in normal controls, benign tumors, early- and late-stage oral melanoma (OM) and oral squamous cell carcinoma (OSCC) as log2 intensities. Asterisks (*) indicate selected proteins involved in networks of protein-chemotherapy drug interactions.
(XLSX)
