# Supplementary figures and images for: Proteomic analysis of canine oral tumor tissues using MALDI-TOF mass spectrometry and in-gel digestion coupled with mass spectrometry (GeLC MS/MS) approaches
Source: PLoS One. 2018 Jul 12;13(7):e0200619. doi: 10.1371/journal.pone.0200619 (PMC6042759; doi:10.1371/journal.pone.0200619)

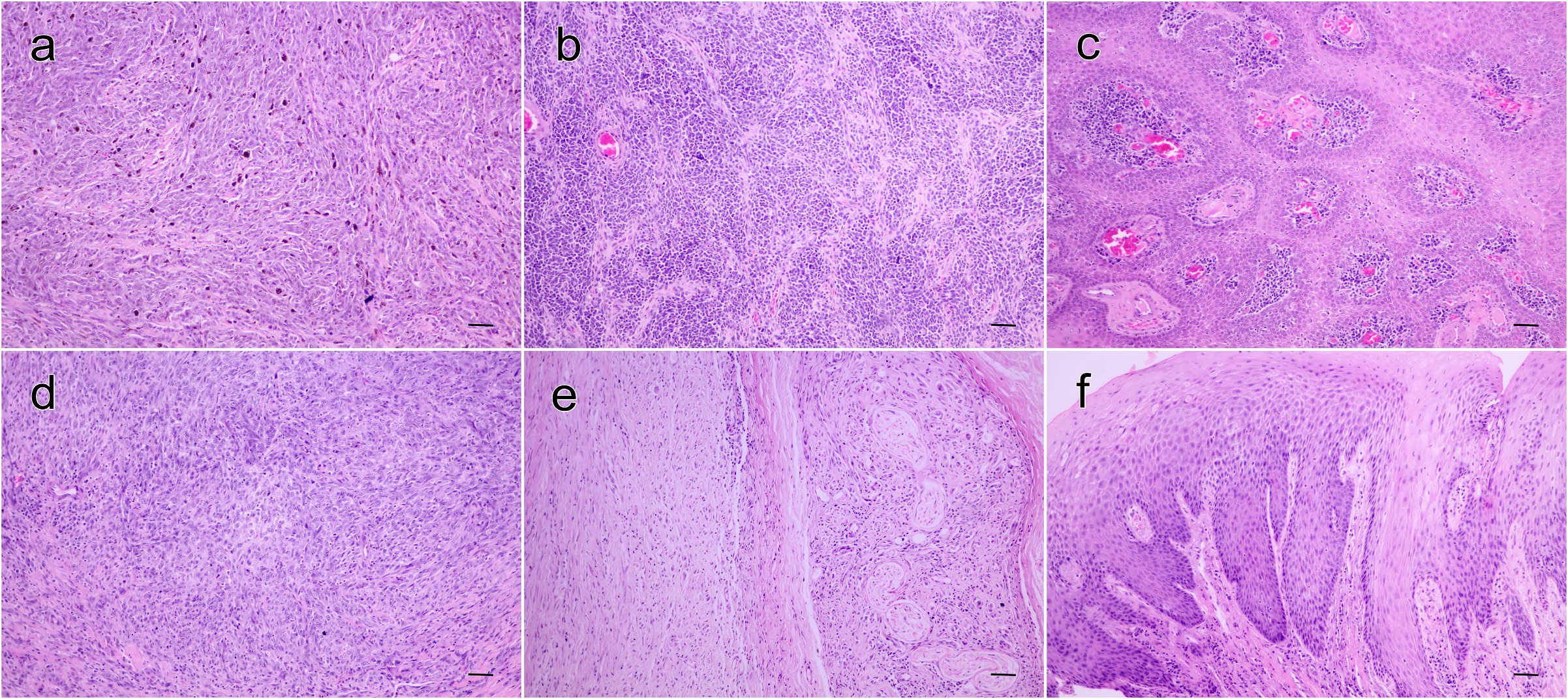

Supplement: S1 Fig — Bar, 50 mm, Inset: Bar, 20 mm. (TIF) [file pone.0200619.s001.tif]
